# Transmission Dynamics, Border Entry Screening, and School Holidays during the 2009 Influenza A (H1N1) Pandemic, China

**DOI:** 10.3201/eid1805.110356

**Published:** 2012-05

**Authors:** Hongjie Yu, Simon Cauchemez, Christl A. Donnelly, Lei Zhou, Luzhao Feng, Nijuan Xiang, Jiandong Zheng, Min Ye, Yang Huai, Qiaohong Liao, Zhibin Peng, Yunxia Feng, Hui Jiang, Weizhong Yang, Yu Wang, Neil M. Ferguson, Zijian Feng

**Affiliations:** Chinese Center for Disease Control and Prevention, Beijing, People’s Republic of China (H. Yu, L. Zhou, L. Feng, N. Xiang, J. Zheng, M. Ye, Y. Huai, Q. Liao, Z. Peng, Y. Feng, H. Jiang, W. Yang, Y. Wang, Z. Feng);; Imperial College London, London, United Kingdom (S. Cauchemez, C.A. Donnelly, N.M. Ferguson)

**Keywords:** transmission, school closure, border screening, influenza A, pandemic (H1N1) 2009, People’s Republic of China, influenza, viruses

## Abstract

Pandemic influenza A (H1N1) 2009 virus spread rapidly around the world in 2009. We used multiple data sources from surveillance systems and specific investigations to characterize the transmission patterns of this virus in China during May–November 2009 and analyze the effectiveness of border entry screening and holiday-related school closures on transmission. In China, age distribution and transmission dynamic characteristics were similar to those in Northern Hemisphere temperate countries. The epidemic was focused in children, with an effective reproduction number of ≈1.2–1.3. The 8 days of national holidays in October reduced the effective reproduction number by 37% (95% credible interval 28%–45%) and increased underreporting by ≈20%–30%. Border entry screening detected at most 37% of international travel–related cases, with most (89%) persons identified as having fever at time of entry. These findings suggest that border entry screening was unlikely to have delayed spread in China by >4 days.

## Abstract 2

Screening delayed spread by <4 days; autumn school holidays reduced the effective reproduction number by ≈40%.

Pandemic influenza A (H1N1) 2009, hereafter referred to as A(H1N1)pdm09, spread rapidly, resulting in millions of cases and ≈18,000 deaths in ≈200 countries (*1*). On August 10, 2010, the World Health Organization (WHO) declared that the world had entered the postpandemic period (*2*). Much has been published about the epidemiology of the pandemic in Western countries (*3**–**9*), but far less has been published about the experience of a large and diverse country, such as the People’s Republic of China. In addition, although many countries adopted so-called early containment strategies, data on their effectiveness are rare (*7**,**10**,**11*).

In response to the evolving global spread of A(H1N1)pdm09 virus infection, China established national surveillance on April 30, 2009. Initially, the country implemented an aggressive containment strategy based on the national pandemic preparedness plan, including isolation of all suspected case-patients in designated hospitals, contact tracing, medical observation of persons exposed to patients with confirmed cases, and border entry screening (Technical Appendix). On May 11, the first case of A(H1N1)pdm09 in mainland China was identified in a traveler returning from the United States (*12*). We report the transmission patterns of A(H1N1)pdm09 in China from that time through November 2009 and analyze the effectiveness of border entry screening and holiday-related school closures on transmission using multiple data sources from surveillance systems and specific investigations.

## Methods

### Sentinel Surveillance for Influenza-like Illness

National sentinel hospital-based surveillance for influenza-like illness (ILI) was launched in China in 2005. This type of surveillance is primarily dedicated to virologic surveillance with a goal of providing information for annual WHO influenza vaccine selection (Technical Appendix). Each week, 193 sentinel hospitals in 30 provinces report the total number of outpatient visits and the number of those patients with ILI by age group to a centralized online system maintained by the Chinese Center for Disease Control and Prevention (China CDC). In addition, respiratory specimens are collected each day from the first or second ILI case-patient who visits each hospital’s outpatient clinic. This collection results in virologic samples from 10–15 respiratory tract specimens per hospital each week. Specimens are sent to 1 of the 62 province- or prefecture-level disease control centers for testing. Laboratory results are reported weekly online to China CDC. These data are collected systematically throughout the year and are an unbiased sample of the timing of influenza activity.

### Individual Case-based Surveillance

During the early containment phase of the 2009 pandemic (until mid-July 2009), an individual case–based surveillance system was implemented. A(H1N1)pdm09 virus infection was added to China’s list of notifiable communicable diseases on April 30, 2009. Persons with suspected A(H1N1)pdm09 infection were identified through active surveillance with border entry screening and medical monitoring of close contacts exposed to confirmed case-patients or through passive reporting by clinicians when those patients sought health care. Any person entering China was required to undergo screening at the border (any point of entry into China from another country or from a neighboring region, such as Hong Kong Special Administrative Region), regardless of border type or travel mode. All patients with suspected A(H1N1)pdm09 virus infection, regardless of its clinical severity, were admitted to designated hospitals for containment (*13**,**14*). Upper respiratory specimens were collected and sent to the national sentinel ILI surveillance network of 62 laboratories for A(H1N1)pdm09 testing by real-time reverse transcription PCR (rRT-PCR) (Technical Appendix). All suspected and laboratory-confirmed cases were reported online within 24 hours to China CDC by public health officers in county-, prefecture-, and province-level disease control centers and clinicians nationwide. Data posted on a standardized reporting card included sex, age, place, overseas travel history, and date of symptom onset.

### Outbreak Surveillance

In accordance with recommendations from the Ministry of Health of China, local disease control centers were asked to investigate all institutional or community outbreaks (e.g., associated with particular schools or shared public transport vehicles) by using the case definition for acute respiratory illness (ARI). Data on all suspected cases, probable cases, and confirmed cases were reported online to China CDC.

### Investigation of Cases Linked to International Travel

In addition, through July 31, a joint team from local disease control centers and China CDC investigated confirmed international travel–related cases (Technical Appendix) to collect detailed epidemiologic information. A standardized questionnaire was used to collect data about international travel histories, date of symptom onset, and reported symptoms on arrival in China. Data on contacts were also obtained. In accordance with Ministry of Health recommendations, all close contacts of confirmed case-patients were quarantined at home or in designated hotels and monitored daily for fever and respiratory symptoms for 7 days after their last exposure to a confirmed case-patient.

We also learned whether the case was detected at the border. Data were not available on how these case-patients entered mainland China (e.g., by air, sea, or land).

### Case Definitions

A case-patient with ARI had fever (temperature >37.3°C), and/or recent onset of >1 of the following: rhinorrhea, nasal congestion, sore throat, or cough. A case-patient with ILI had a body temperature >38°C with either cough or sore throat in the absence of an alternative diagnosis. A person with a suspected case of A(H1N1)pdm09 virus infection had ARI and 1 of the following: illness onset within 7 days after travel to an area with >1 confirmed A(H1N1)pdm09 cases or within 7 days after close contact with a confirmed case-patient. A person with a confirmed case had ARI and laboratory evidence of A(H1N1)pdm09 virus infection diagnosed by rRT-PCR of respiratory specimens. A person with a probable case had ARI that was epidemiologically linked to a patient with a confirmed case. On the basis of information about overseas travel and any identified links to other known case-patients, all reported confirmed cases were classified as international travel–related cases, individual domestic cases, and institutional or community outbreaks.

### Change in Surveillance Strategy

By mid-August 2009, as A(H1N1)pdm09 activity expanded, the national surveillance strategy changed from individual case-based surveillance to identification of hospitalized patients who required medical treatment for complications, identification of outbreaks, and ongoing routine sentinel ILI surveillance. Only patients who required hospital care were admitted; patients with milder infection were cared for at home.

### Statistical Analysis

The serial interval of an infectious disease is defined as the time between onset of symptoms in an index patient and onset of symptoms in an infected contact. We analyzed data on transmission among the first 47 identified clusters we investigated, each with a single index case, to estimate the serial intervals associated with 60 infected contacts.

We estimated the incubation period distribution using data from the 22 persons with identified single-day exposures and the 35 persons with identified multiple-day exposure intervals (Technical Appendix Table 2), excluding 3 persons with exposures implying incubation periods of >20 days (Technical Appendix). We report the posterior median and 95% credible interval (CrI) of the mean and SD of the incubation period.

Doubling times in case numbers were estimated from the epidemic curve of weekly ILI incidence attributable to A(H1N1)pdm09 virus infection, obtained by multiplying raw ILI data by the weekly proportion of ILI case-patients who tested positive for A(H1N1)pdm09 virus. Those estimates, along with the evidence-based assumption that the generation time of influenza A(H1N1)pdm09 had a mean of 2.6 days and an SD of 1.3 days (*3**,**5**,**6**,**15**,**16*) (consistent with data analyzed on the serial interval), were used to estimate the effective reproduction number of A(H1N1)pdm09 virus infections in China. A simple epidemic model was fitted to the A(H1N1)pdm09 virus–attributable ILI case curve on the calendar weeks before and after the National Day Holiday (October 1–8) to estimate the effect of holidays on effective reproduction numbers and reporting rates. The model is based on the observation that numbers of cases increase at a rate that is a function of the reproduction number and the generation time of the disease (*17*). From the rate of growth of case numbers observed in the epidemic and the generation time of influenza A(H1N1)pdm09, the model can be used to derive the reproduction number of the disease for different intervals. In the past, this approach has been used to estimate the reproduction number of 1918 pandemic influenza in US cities (*18*).

International travel–related case-patients who had symptoms on arrival were classified as either “having fever” or “without fever but having respiratory symptoms” (Technical Appendix). Frequency tables (with χ^2^ tests) were constructed to examine the univariate associations between the probability of detection at the border and patients’ characteristics. Univariable and multivariable logistic regression models were used to examine potential predictors of the probability of detection at the border (fever on arrival, time between onset and arrival, age group, and province) individually and simultaneously (i.e., using univariable and multivariable regression models, respectively) and to quantify their effects.

## Results

### Confirmed Cases

**Figure 1:**
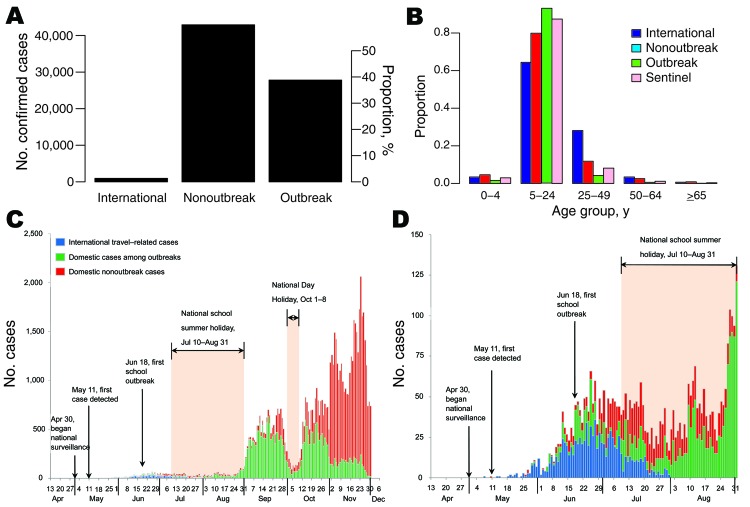
Confirmed cases of influenza A(H1N1)pdm09 virus infection, People’s Republic of China, 2009. A) Number and proportion of confirmed A(H1N1)pdm09 cases by type (international travel–related cases, nonoutbreak cases, outbreak cases). B) Age distribution of patients with confirmed cases of A(H1N1)pdm09 infection gathered from different data sets. C, D) Number of confirmed A(H1N1)pdm09 cases by date of illness onset during May–August 2009 (C) and May–November 2009 (D) from case-based surveillance and outbreak investigations.

During May 7–November 30, 2009, a total of 71,665 persons with confirmed A(H1N1)pdm09 virus infection were reported to China CDC. Of those, 932 (1%) were related to international travel; 27,806 (39%) cases were detected during domestic outbreak investigations; and 42,917 (60%) were domestic nonoutbreak cases (Figure 1, panel A). The first case-patient was a traveler who returned from the United States with illness onset on May 7; the first domestic case-patient had symptom onset on May 10 (Figure 1, panels C, D). The origin of reported cases slowly shifted: most cases were international travel related until early June; in June, roughly half were international travel related and the other half were domestic; in July, most cases were domestic (Figure 1, panel C; Technical Appendix Figure 2). The last known international travel–related case was reported on July 31, after which intensive border entry screening was gradually reduced. Irrespective of the type of case, persons 5–24 years of age were most affected, with the proportion of cases ranging from 64% in international travelers to 94% in outbreak cases (Figure 1, panel B). The proportion of persons 25–49 years was <12% in all categories, except international travel–related cases, for which it was 28% (likely because persons 25–49 years were overrepresented among international travelers).

**Figure 2:**
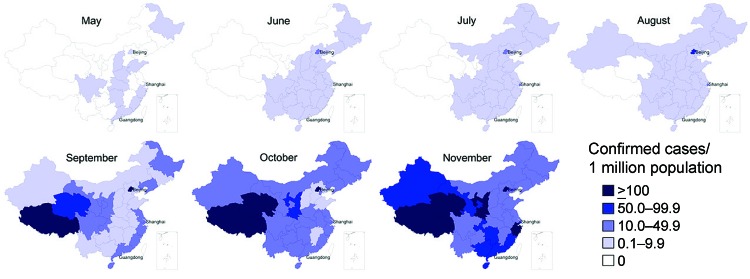
Incidence of confirmed cases of influenza A(H1N1)pdm09 virus infection per 1,000,000 inhabitants, by month and province, People’s Republic of China, May–November 2009.

The infection spread rapidly throughout China; 11 provinces (containing many of the most globally connected cities) reported confirmed cases in May, and all but 5 western provinces reported cases in July (Figure 2). By September, all provinces reported confirmed cases. Geographic variation occurred in the incidence of confirmed cases per 1 million persons throughout the epidemic, but how much this variation was caused by surveillance system variation (e.g., differences in access, use of health care, in laboratory capacity) is difficult to determine.

### Sentinel ILI Surveillance

**Figure 3:**
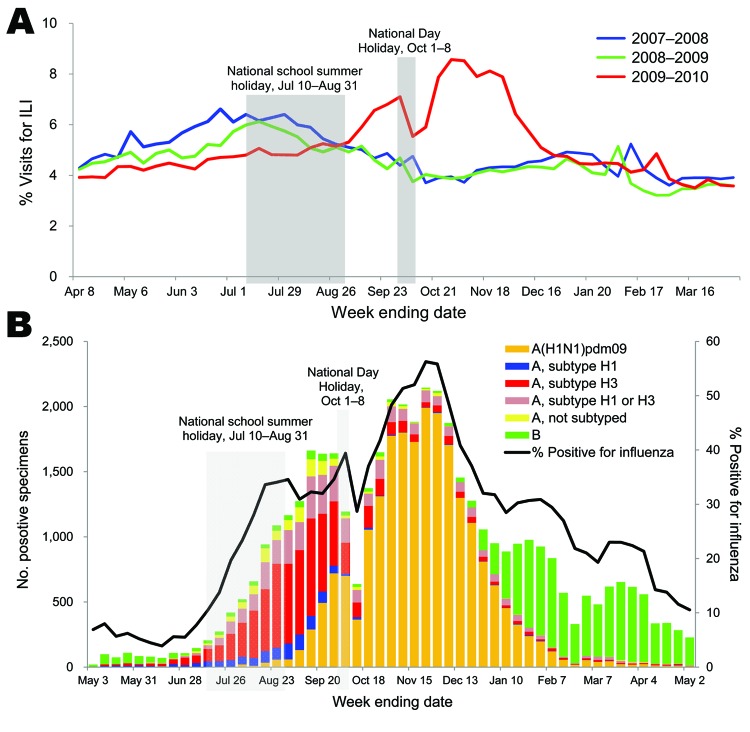
Sentinel surveillance for influenza-like illness (ILI) and virologic surveillance, People’s Republic of China, 2007–2010, A) Weekly percentage of visits for ILI, sentinel ILI surveillance, People’s Republic of China, 2007–08 through 2009–10. B) Number and percentage of specimens positive for influenza, by week of specimen collection during sentinel ILI surveillance in China, May–November 2009.

The percentage of visits for ILI from sentinel surveillance increased slowly from May 2009 through the end of August 2009, although the percentage was lower than that observed during the same months in 2007 and 2008 (Figure 3, panel A). In September 2009, ILI activity increased substantially and was higher than in the 2 previous seasons. ILI activity decreased sharply during the National Day Holiday, then rebounded at the end of the holiday period. Similar fluctuations were observed for other influenza viruses (Figure 3, panel B). The number and proportion of influenza-positive cases from sentinel ILI surveillance increased stably from May 2009 onward; A(H1N1)pdm09 became the predominant strain at the end of September and subsequently declined after early December.

### Serial Interval and Incubation Period

**Figure 4:**
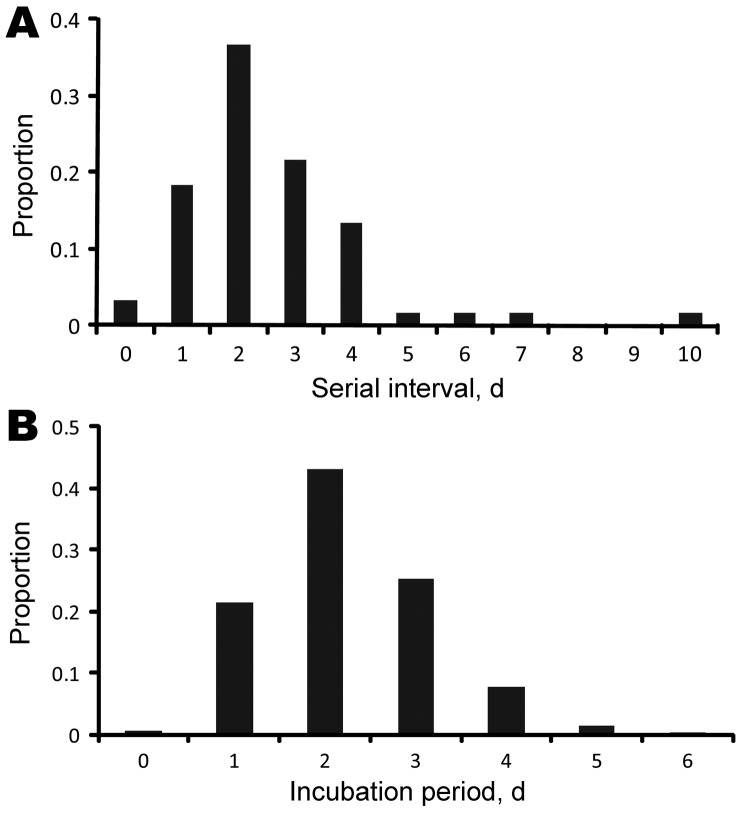
Natural history of influenza A(H1N1)pdm09 virus infection, People’s Republic of China, 2009. A) Distribution of serial intervals among clusters of cases, each with a single index case. B) Incubation period distribution estimated from the 22 persons with identified single-day exposures and the 35 persons with identified multiple-day exposure intervals.

In the household setting, the average serial interval was 2.6 days (95% CI 2.2–3.0 days; Figure 4, panel A). Similar results were obtained in the analysis restricted to data from the 38 clusters in which the single index case-patient transmitted infection to a single contact. The incubation period had a mean of 2.2 days (95% CrI 1.9–2.5 days) and an SD of 1.0 days (95% CrI 0.8–1.2 days) (Figure 4, panel B).

### Transmissibility and Effect of Holidays on Spread

**Figure 5:**
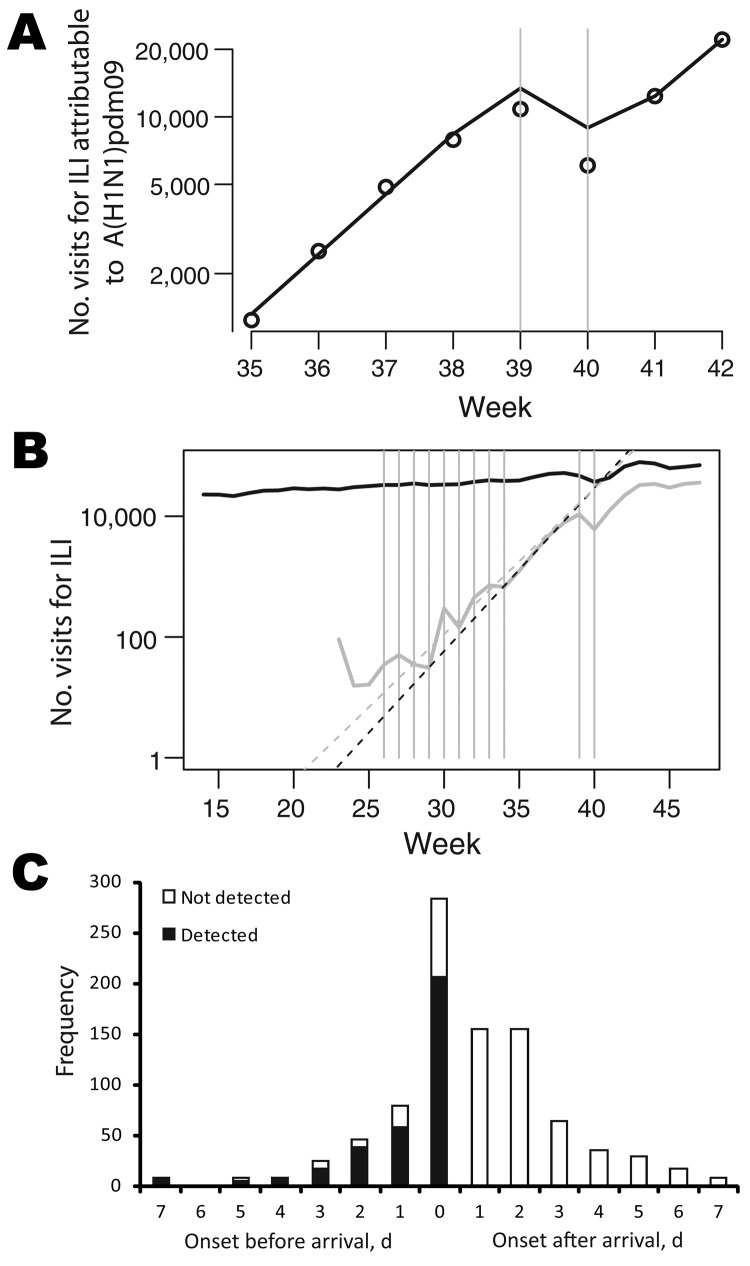
Effects of school holidays and border entry screening on influenza A(H1N1)pdm09 virus infections, People’s Republic of China, 2009. A) Observed (black points) and predicted (solid line) number of visits for influenza-like illness (ILI) attributable to A(H1N1)pdm09 from week 35 (ending September 6) through week 42 (ending October 25). National Day Holiday occurred from Thursday, October 1 (week 39), through Thursday, October 8 (week 40). A simple epidemic model was fitted to data for calendar weeks before and after the National Day Holiday (gray bars) so that potential changes in reporting rates during holidays could be estimated. B) Number of visits for ILI. The black solid line shows raw numbers of visits for ILI; the gray solid line shows numbers corrected by the weekly proportion of ILI cases that are positive for A(H1N1)pdm09 virus. Gray dashed line shows growth rate in July-August. Black dashed line shows growth rate during the first 3 weeks of September. Gray bars indicate holiday periods. C) The distribution of intervals between symptom onset and arrival in China among confirmed international travel–related case-patients (N = 932).

We estimated that the effective reproduction number changed from 1.25 (95% CrI 1.22–1.28) before the National Day Holiday (August 31–September 30) to <1 during that holiday (0.79; 95% CrI 0.69–0.90) and back to 1.23 (95% CrI 1.15–1.32) after that holiday (October 7–October 25) (Figure 5, panel A; Table 1; Technical Appendix Table 1). The National Day Holiday was therefore found to reduce the effective reproduction number by 37% (95% CrI 28%–45%). Our model also predicted that underreporting had increased by 19% (95% CrI 6%–31%) and 32% (95% CrI 11%–48%), respectively during the first and second calendar weeks of the National Day Holiday. However, the 8-week summer school holiday appeared to have had a limited effect on transmission as measured by A(H1N1)pdm09 virus–attributable ILI incidence, in contrast to what was observed in other countries, such as the United Kingdom (*19*). The doubling time during the summer school holiday (8.7 days during July 13–August 30) was similar to that observed in the month after schools reopened in September (7.1 days) (Figure 5, panel B). Using the rate of growth observed during July–August (Figure 5, panel B), we extrapolated the A(H1N1)pdm09 virus–attributable ILI case curve back in time and inferred that the first sentinel-detected ILI case caused by A(H1N1)pdm09 virus occurred in China in week 19 (May 11–17), near the date when the first imported case was detected (May 11).

**Table 1:** Reproduction numbers obtained by fitting a simple epidemic model to numbers of influenza-like illness cases attributable to pandemic influenza A (H1N1) 2009 virus, People’s Republic of China, September–October 2009*

Interval	Mean (95% credible interval)
School term 1 (Sep 6–Oct 1)	1.25 (1.22–1.28)
Holidays (Oct 1–8)†	0.79 (0.69–0.90)
School term *2* (Oct 9–25)	1.23 (1.15–1.33)

*Fitted on data for the calendar weeks before and after the National Day Holiday. †Period of National Day Holiday. Details can be found in Technical Appendix Table 1.

### Effectiveness of Border Entry Screening

International travel–related cases were detected either at the border or later by contact tracing and passive case finding within the country. Overall, 37% of international travel–related cases ever detected were detected at the border. The timing of onset of symptoms affected the probability of detection by symptom screening at the border. Half (468/932) of international travel–related case-patients ever detected had onset of symptoms >1 days after arrival (Figure 5, panel C).

Among international travel–related case-patients who had symptoms on arrival, those with fever were significantly more likely to be detected at the border. The percentage of patients detected at the border was as follows: 76% for those with fever, 63% for those without fever but with respiratory symptoms (χ^2^ 4.41; df 1; p = 0.036; n = 464). Overall, 74% of persons ever detected with symptom onset on or before the day of arrival were identified at the border. Multiple logistic regression modeling showed a significant interaction (p = 0.023) between whether a case-patient had a fever on arrival and the time between symptom onset and arrival (stratified by those with onsets 0 or 1 day before arrival and those with onsets >1 day before arrival; Table 2, Table 3). Thus, if a case-patient had a fever on arrival, then the time since onset was irrelevant. Similarly, the odds ratios (ORs) were similar for those with fever on arrival and onset 0 or 1 day before arrival (OR 1.80) relative to those with no fever and onset 0 or 1 day before arrival) and those with fever on arrival and onset >1 day before arrival (OR 1.91 relative to those with no fever and onset 0 or 1 day before arrival) (Table 3). However, among persons who did not have a fever on arrival, those who had been ill longer before arrival (>1 day) were more likely to be detected at the border (the percentage of detection = 83%, Table 2; OR 2.36, Table 3). After adjusting for these effects, neither age group nor province affected the probability of a case being detected at the border.

**Table 2:** Percentage of case-patients detected by symptom status and interval between symptom onset and arrival among international travel–related case-patients who were symptomatic on arrival, People’s Republic of China, 2009

Onset	Fever on arrival, %	No fever on arrival, %	Total, %
0 or 1 d before arrival	75, n = 329	49, n = 35	73, n = 364
>1 d before arrival	76, n = 76	83, n = 24	78, n = 100
Total	76, n = 405	63, n = 59	74, n = 464

**Table 3:** Potential predictors of the probability of detection at the border of international travel–related pandemic influenza A (H1N1) 2009 infection, People’s Republic of China, 2009*

Model	OR (95% CrI)	p value
Univariable		
Fever on arrival	1.84 (1.04–3.27)	0.042
Onset of symptoms >1 d before arrival	1.05 (0.91–1.22)	0.288
Age group, y†		0.126
0–4	1	
5–24	1.67 (0.55–5.03)	
25–49	1.12 (0.36–3.50)	
50–64	0.58 (0.13–2.69)	
Province‡		0.400
44	1	
31	1.05 (0.49–2.23)	
35	0.66 (0.30–1.45)	
Multivariable§		
Fever on arrival	1.80 (1.26–2.57)	0.001
Onset of symptoms >1 d before arrival	2.36 (1.18–4.73)	0.016
Interaction between fever on arrival and onset of symptoms >1 d before arrival	0.45 (0.22–0.89)	0.023

*OR, odds ratio; CrI, credible interval. †n = 463. Only 1 such person was >64 y of age, and this person was excluded from this analysis. ‡n = 302. Only 3 provinces had >40 persons so only they are included here. §Likelihood ratio–based p value for the global hypothesis that all ORs equal 1 (3 df 0.008). The deviance (that is minus twice the log likelihood) for the multivariable model presented was 520.6, whereas the deviance for the model with only an intercept (i.e., no effects of fever or time from onset to arrival) was 532.5.

## Discussion

We described transmission patterns of A(H1N1)pdm09 virus infection in China during 2009 by using multiple epidemiologic data collected from surveillance and investigations. The age distribution and transmission dynamic parameters, including incubation period and serial interval, are consistent with those observed in other countries (*3**–**6*).

We can put an upper boundary only on the effectiveness of the border screening adopted early in the pandemic because data are available only on cases detected (either at the border or later through case-tracing), rather than missed cases. Given that travelers with mild illness or subclinical infection might not seek health care, a substantial proportion of international travel–related cases were likely never detected and therefore did not appear in our dataset. Hence, the proportion of imported cases that were detected at the border was, at most, 37%. Assuming the doubling time of the global epidemic in May was similar to that seen in China during July–August (8.7 days), and if the border screening reduced transmission from case-patients with imported cases by 37% (i.e., isolation of detected cases was 100% effective), the epidemic in China would have been delayed by 4 days (the additional time taken for cumulative imported cases to reach the level they would have reached in the absence of border controls). Thus, border controls likely delayed the epidemic by only a few days, even assuming few imported cases were missed altogether. This conclusion is supported by the observation that the trajectory of the epidemic in China appears relatively similar to that seen in the United Kingdom, another country to which infection had spread early in May but that did not employ border screening. Clearly, symptom-based border screening cannot detect infections among persons who are asymptomatic on arrival.

Our analysis suggests that the October national holiday might have reduced transmission by as much as 37% and reporting by ≈20%–30%. The National Day Holiday in China is similar in scope to the Christmas holiday in Western countries, with all kindergartens, schools, and universities and many businesses being closed. Most citizens leave their routine work, but festivals, mass gatherings, and travel occur during this period. However, the Summer School Holiday appears to have reduced transmission by a minimal amount (no more than 3% reduction in the effective reproduction number), in contrast to the large drop seen in other countries such as the United Kingdom. Why this discrepancy exists are unclear but might relate to the much more frequent use of collective childcare and summer camps and schools by Chinese parents during summer holidays than is typical in many European countries. In addition, seasonal factors that can limit transmission in temperate countries in summer might have had more limitedly affected the southern subtropical provinces of China.

The effective reproduction number for A(H1N1)pdm09 in China ranged from 1.2 to 1.3, which is consistent with that observed in other countries, although in the lower range. In comparison, the effective reproduction number was ≈1.4 in the United Kingdom in June–July 2009 (*19*). Because the proportion of the population <15 years of age is similar in both countries, demographic differences would not appear to explain these differences. However, spatial heterogeneity in the efficiency of spread and desynchrony between the epidemics in different regions of China might lead to the underestimation of transmissibility on a national scale. This remains a topic for future analysis. We relied on A(H1N1)pdm09 virus–attributable ILI incidence to estimate the epidemic growth rate because the proportion of ILI case-patients who tested positive for influenza increased substantially during the pandemic (Figure 3, panel B). As a consequence, the growth rate of the ILI incidence curve underestimates the epidemic growth rate (Figure 5, panel B). A similar approach was used by Baguelin et al. (*19*).

Our study has several limitations, which are inevitable, given that many of the data were collected as part of public health control rather than specifically to inform epidemiologic characterization of the pandemic. Case-based surveillance established by many countries in the early phase of the pandemic was critical to monitor early emergence and extent of geographic spread. However, in retrospect, those systems were not able to monitor the growth in case numbers over time because the ability to identify cases and conduct outbreak investigations could quickly be limited by saturated resources, for example, laboratory diagnostic capacity. Furthermore, the change from reporting individual cases regardless of clinical severity to reporting hospitalized cases likely affected the reporting rate of confirmed A(H1N1)pdm09 cases during mid-July and mid-August. In contrast, sentinel surveillance was not influenced by the change in case-based surveillance during the pandemic. However, for a country as large and diverse as China, some geographic variability is almost unavoidable in the quality of the surveillance system and capacity of health care system. This variability could make comparison of incidence levels by geographic zone somewhat difficult.

Improving and monitoring the homogeneity of the Chinese surveillance and health care system are challenging, yet vital, tasks to improve the monitoring of future pandemics. The effects of other interventions also need to be assessed, for example, strict case isolation, contact tracing, and medical observation, which might have helped delay the spread at early containment stage of the pandemic.

Thus, the overall picture of the epidemiology and transmission dynamics of A(H1N1)pdm09 that emerges from the surveillance data is comparable to that in many European countries and the United States. Border entry screening during the influenza pandemic delayed spread in China by a few days, at most, but the autumn school holidays reduced the effective reproduction number by ≈40%.

## Supplementary Material

Technical Appendix Influenza A(H1N1)pdm09, People's Republic of China, May–December 2009.
