# Supplementary material for: Transmission Dynamics, Border Entry Screening, and School Holidays during the 2009 Influenza A (H1N1) Pandemic, China
Source: Emerg Infect Dis. 2012 May;18(5):758–66. doi: 10.3201/eid1805.110356 (PMC3358060; doi:10.3201/eid1805.110356)
Supplement: Technical Appendix — Influenza A(H1N1)pdm09, People's Republic of China, May–December 2009. [file 11-0356-Techapp_13p.pdf]

Article DOI: http://dx.doi.org/10.3201/eid1805.110356

Transmission Dynamics, Border Entry Screening, and School Holidays during the 2009 Influenza A (H1N1) Pandemic, China

Contents

1 Containment interventions

1.1 Border entry screening

1.2 Case management

1.3 Medical observation of close contacts

2 Definitions

2.1 International-travel-related case

2.2 Close contact

3 Background information on national sentinel ILI surveillance

4 Laboratory testing

5 Derivation of exponential growth rates, doubling times and reproduction numbers

6 Fitting a piecewise exponential growth model to H1N1 attributable ILI visits

7 Impact of holidays on the epidemic curve of confirmed cases

8 References

1 Containment interventions

1.1 Border entry screening

From April 25 to mid of August, upon international arrival in China, all travelers via air, sea and land originated from a country with confirmed H1N1pdm infection were requested to complete a health declaration form at the entry points, and were screened for fever by hand wands or fixed-position infrared thermal scanner. Travellers who declared any one of following symptoms: fever, rhinorrhea, nasal congestion, sore throat, cough, headache, myalgia, dyspnea, or diarrhea in the form, or with fever detected by thermal scanner were evaluated by medical personnel and their body temperature was taken using mercuric thermometer. Any patient diagnosed with Acute Respiratory Illness (ARI) was isolated immediately at a local designated hospital for quarantine and tested for H1N1pdm virus infection by real-time reverse transcription polymerase chain reaction (rRT-PCR).

1.2 Case management

Early on, all suspected cases regardless of clinical severity were immediately admitted to designated hospitals and placed in a private room or a room with negative pressure, if available, for respiratory isolation. From mid-August 2009 onward, hospitalization was based on clinical judgment and mild cases were recommended to self isolate at home until 24h after clinical recovery.

1.3 Medical observation of close contacts

Up to July 8, close contacts of confirmed cases were identified as soon as possible and quarantined at home or in designated hotels, and monitored daily for fever and respiratory symptoms for 7 days after their last exposure to a confirmed case. The Ministry of Health of China did not recommend antiviral chemoprophylaxis for close contacts.

2 Definitions

2.1 International-travel-related case

For analysis purpose in this study, an international-travel-related case was defined as a case who had travelled to a country with confirmed H1N1pdm infection within 7 days before illness onset, while for those without such overseas travel history were defined as domestic cases.

2.2 Close contact

A close contact was defined as a person known to have been within 2 meters of a confirmed case-patient for any length of time during the case's infectious period, including household and social contacts, and health care workers who were assessed to have used suboptimal personal protective equipment. For airplane passengers and crew, a close contact was defined as any crew member who had provided face-to-face service to a confirmed case, or as any passenger seated in the same row or within three rows in front of or behind a confirmed case, adapted from WHO guidance (*1*). The infectious period for a confirmed case was defined to be one day prior to and through 7 days after illness onset or 24 h after resolution of symptoms, whichever was longer (*2*).

3 Background information on national sentinel ILI surveillance

The national sentinel hospital-based Influenza Like Illness (ILI) surveillance network was established in 2000, subsequently was expanded to 30 provinces and run stably using a standard ILI case definition (body temperature ≥38°C with either cough or sore throat in the absence of an alternative diagnosis) since October 2005. Each week, 193 sentinel hospitals of 30 provinces with exception of Tibet within the network report the total number of outpatient and/or

emergency department visits and the number of those patients with ILI by age group to a centralized on-line system maintained by China CDC. In addition, a subset of 10-15 respiratory specimens (nasopharyngeal swabs) were collected from ILI cases firstly arrived on average each week, on the day when they presented to the outpatient and/or emergency department, and placed in sterile viral transport medium for influenza virus testing following a standard protocol (*3*). The sample was inoculated into Madin-Darby canine kidney (MDCK) cells and/or specific pathogen free (SPF) chicken embryo for virus isolation. Hemagglutination inhibition (HI) and/or real-time reverse transcription polymerase chain reaction (rRT-PCR) assay were performed to identify types and subtypes of influenza virus as appropriate. These assays were performed in biosafety level (BSL) 2 facilities of 31 provincial and 32 prefecture level Centers for Disease Control and Prevention, and quality controlled by the National Influenza Center (NIC) of the China CDC. All reagents, primers and probes were provided by the NIC, China CDC. Such surveillance activities were implemented year-round in 99 hospitals of 15 Southern provinces in subtropical or tropical region (below 34° latitude) and 22 hospitals of three Northern provinces (Tianjin, Liaoning, and Gansu) in temperate regions, while running in winter season from October to next March for remaining 72 hospitals of 12 Northern provinces in temperate regions. In response to evolving pandemic H1N1, all 193 sentinel hospitals were asked to implement year-round since May 2009, and the network was expanded to all 411 provincial and prefecture level CDCs and 556 hospitals around the country since July 2009. We only included surveillance data from 193 sentinel hospitals and 62 laboratories for final analysis, given the data quality and expected suboptimal capacity among additional sentinel ILI surveillance network (additional 363 hospitals and 349 laboratories) established at speed as part of China’s outbreak response.

4 Laboratory testing

Respiratory specimens (nasal, throat, and nasopharyngeal swabs) were collected from suspected H1N1pdm cases and placed in sterile viral transport medium for H1N1pdm virus testing following a standard protocol (*4*). RNA was extracted from specimens using the RNeasy Mini Kit (Qiagen, Valencia, CA) per the manufacturer's protocol and tested by rRT-PCR with H1N1pdm-specific primers and probes following the WHO protocol (*5*). These assays were performed in biosafety level (BSL) 2 facilities of National Sentinel Influenza Surveillance Network, and quality controlled by NIC of the China CDC.

5 Derivation of exponential growth rates, doubling times and reproduction numbers

Denote n_t the number of cases on day t. Under the assumption that the number of cases grows exponentially in time interval $[t_1, t_2]$,

$$n_t = n_{t_1} \exp\{r.(t - t_1)\} \qquad (1)$$

where r is the exponential growth rate. Equation (1) is equivalent to:

$$\log(n_t) = \log(n_{t_1}) + r.(t - t_1) \qquad (2)$$

In practice, the exponential growth rate r can be estimated on time interval $[t_1,t_2]$ via standard least-square fitting. It is equal to the coefficient of the linear regression of $\log(n_t)$ on $(t - t_1)$:

$$\mathrm{r} = \frac{\mathrm{cov}(\{\log(n_t)\}_{t\in[t_1,t_2]},[t_1,t_2]}{\mathrm{var}([t_1,t_2])} \qquad (3)$$

The doubling time d is such that $n_{t+d} = 2.n_t$

With equation (1): $n_t.\exp(r.d) = 2.n_t$

After simplification, the doubling time d is found to be:

$$\mathrm{d} = \frac{\log(2)}{r} \qquad (4)$$

For a Gamma-distributed generation time, the reproduction number is given by:

$$R = \left(1 + \frac{r}{b}\right)^a \qquad (5)$$

where a and b are the parameters of the Gamma distribution ($a = \mu^2/\sigma^2$ and $b = \mu/\sigma^2$ where μ and σ are the mean and standard deviation of the distribution, respectively) (*6*). Here, we make the evidence-based assumption that the generation time of 2009 H1N1pdm influenza has mean μ = 2.6 days and standard deviation σ = 1.3 days (*7–11*).

6 Fitting a piecewise exponential growth model to H1N1-attributable ILI visits

We fit a piecewise exponential growth model to H1N1-attributable ILI visits from week 35 (ending September 6th) to week 42 (ending October 25th) in order to assess the impact of National Day Holiday, that took place from Thursday October 1st (week 39) to Thursday October 8th (week 40), on growth rates, reproduction numbers as well as reporting rates.

Denote I_t the number of cases with symptoms onset at time t. We make the assumption that I_t grows exponential with an exponential growth rate $r(t)$ that is a piecewise function of time t:

$$I_t = I_0 \exp\{-\int_0^t r(u)\,du\}$$

where I_0 is the number of cases with onset at time 0 (defined as the start of week 35) and

$$r(t) = \begin{cases} r_{S1} & \text{if } t < \text{Oct 1st (school term)} \\ r_H & \text{if Oct 1st} \le t \le \text{Oct 8th (holidays)} \\ r_{S2} & \text{if Oct 8th} < t \text{ (school term)} \end{cases}$$

The number of cases with onset in time interval $[t_1,t_2]$ is:

$$U([t_1,t_2]) = \int_{t_1}^{t_2} I_t dt$$

$$U([t_1,t_2]) = \mathrm{I}_{t_1} \int_{t_1}^{t_2} \exp\{-\int_{t_1}^{t} r(u)\, du\} dt$$

Furthermore, we make the assumption that the observed number m_k of cases with onset in week k has a negative binomial distribution with mean U(week k) and size σ (i.e. variance is U(week k)+ $U(\text{week } k)^2/\sigma$).

Parameter vector $\theta = \{rS_1, r_H, rS_2, \sigma\}$ is estimated by fitting the model to H1N1-attributable ILI case curve for calendar weeks before and after the National Day Holiday, that is for weeks 35-38 and 41-42. In the baseline analysis, data for the 2 weeks on which the National Day Holiday took place were not used in the fitting procedure in order to estimate potential changes in reporting rates during those 2 weeks (Figure 5, panel A in the main text; Technical Appendix Table 1). In a sensitivity analysis, we also computed estimates obtained when data from the 2 calendar weeks when the National Day Holiday took place were used in the fitting procedure (Technical Appendix Figure 1; Technical Appendix Table 1).

Inference was done in a Bayesian setting, with flat priors specified for all parameters. Markov chain Monte Carlo sampling was used to explore the joint posterior distribution of the parameters, derive the posterior median and 95% Credible Intervals (*12*).

Equations (4) and (5) were used to derive estimates of doubling times and reproduction numbers from those of the exponential growth rates.

7 Impact of holidays on the epidemic curve of confirmed cases

The succession of holidays and school terms affected the incidence of confirmed cases reported through individual case-based surveillance (Figures 1c and SI3). There was a substantial reduction in the incidence of confirmed cases during the National Day Holiday. In addition, a sharp increase in case numbers was observed when schools reopened, with a very short doubling time of 2.3 days in the time interval August 31st-September 4th and of 2.4 days in the time interval October 9th-11th (Technical Appendix Figure 3). However, it is likely that part of those fluctuations were due to a change in the intensity of surveillance/reporting since once schools had reopened, growth rates quickly reduced to lower values (doubling time of 32.3 days between September 4th-27th and of 37.5 days between October 12th-26th).

Technical Appendix Table 1: Posterior median (95% Credible Intervals) of the exponential growth rates and reproduction numbers obtained by fitting a piecewise exponential growth model to H1N1-attributable ILI case numbers from week 35 (ending September 6th) to week 42 (ending October 25th). The National Day Holiday, took place from Thursday October 1st (week 39) to Thursday October 8th (week 40).

	Baseline fit – calendar weeks when holidays took place are not used in fitting procedure	Sensitivity analysis – calendar weeks when holidays took place are used in fitting procedure
**Growth rate**		
- School term *S1* (up to Oct 1st) r_{S1}	0.088 (0.078, 0.098)	0.083 (0.070,0.094)
- Holidays *H* (Oct 1st-8th) r_H	-0.089 (-0.041, -0.138)	-0.123 (-0.166,-0.076)
- School term *S2* (after Oct 8th) r_{S2}	0.083 (0.053,0.113)	0.113 (0.086,0.141)
**Reproduction number**		
- School term *S1* (up to Oct 1st) R_{S1}	1.25 (1.22,1.28)	1.23 (1.19, 1.27)
- Holidays *H* (Oct 1st-8th) R_H	0.79 (0.69,0.90)	0.72 (0.63, 0.82)
- School term *S2* (after Oct 8th) R_{S2}	1.23 (1.15, 1.33)	1.33 (1.24, 1.42)
**Initial number of cases I_0**	114 (97, 136)	121 (97,158)

Technical Appendix Table 2: Overview of the data on 2009 H1N1 incubation periods based on periods of exposure to index cases. Each row represents the number (n) of individuals with a particular incubation period (red) or a particular range of possible incubation periods (blue).

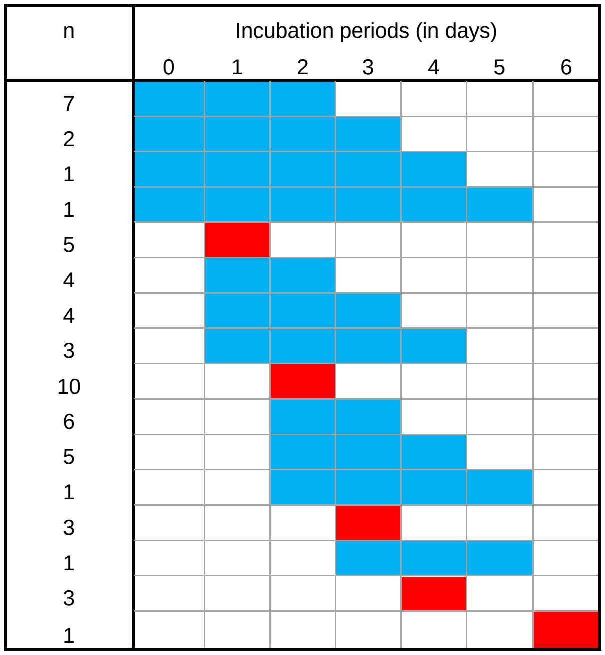

Technical Appendix Figure 1: Observed (black points) and predicted (red line) number of H1N1-attributable ILI visits from week 35 (ending September 6th) to week 42 (ending October 25th). Same as Figure 5a in the paper but when data from the calendar weeks when holidays take place are used in the fit.

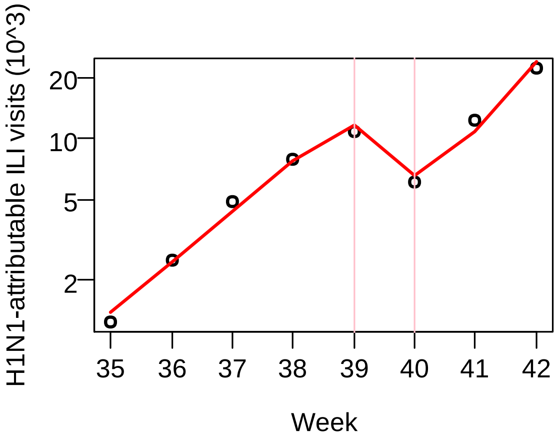

Technical Appendix Figure 2: Daily and cumulated proportion of international-travel-related cases in the early phase of the pandemic.

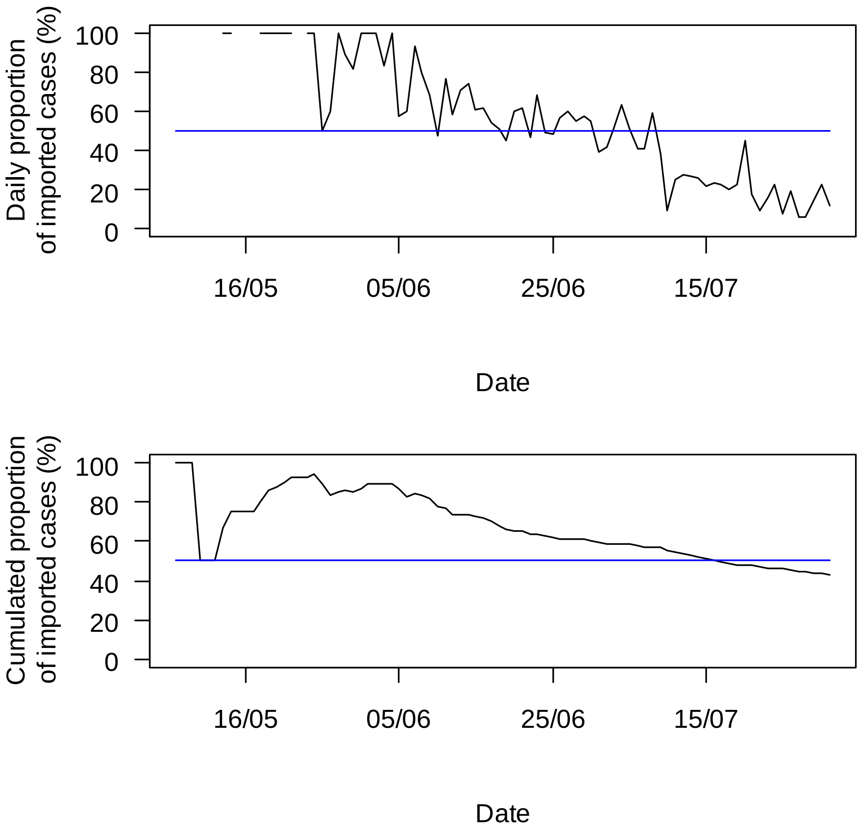

Technical Appendix Figure 3: Daily number of confirmed H1N1pdm cases (log-scale) derived from case-based surveillance from August 25th to November 15th. Pink bars indicate holiday periods. The red line shows the expected number of cases extrapolated from the school-term time period September 4th-September 27th. The green and blue lines show the sharp growth in case numbers observed when schools reopen on September 1st (green) and on October 9th (blue).

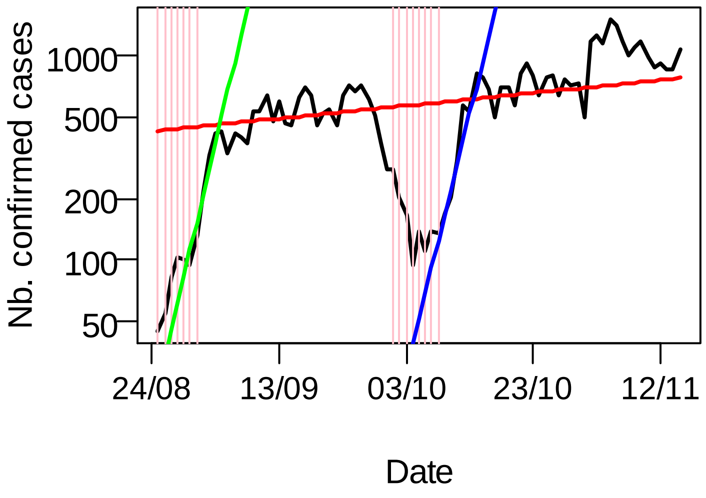

8 References

1. WHO (2010) WHO technical advice for case management of Influenza A (H1N1) in air transport.

2. CDC. (2009) Interim Guidance on Infection Control Measures for 2009 H1N1 Influenza in Healthcare Settings, Including Protection of Healthcare Personnel.

3. Chinese-CDC Influenza sentinel surveillance protocol (2005-2010). August 19, 2005 ed.

4. CDC Interim Guidance on Specimen Collection, Processing, and Testing for Patients with Suspected Swine-Origin Influenza A (H1N1) Virus Infection.

5. WHO CDC protocol of realtime RTPCR for swine influenza A (H1N1).

6. Fraser C, Donnelly CA, Cauchemez S, Hanage WP, Van Kerkhove MD, Hollingsworth TD, et al. Pandemic potential of a strain of influenza A (H1N1): early findings. Science. 2009;324:1557–61. PubMed http://dx.doi.org/10.1126/science.1176062

7. Cauchemez S, Carrat F, Viboud C, Valleron AJ, Boelle PY. A Bayesian MCMC approach to study transmission of influenza: application to household longitudinal data. Stat Med. 2004;23:3469–87. PubMed http://dx.doi.org/10.1002/sim.1912

8. Cauchemez S, Donnelly CA, Reed C, Ghani AC, Fraser C, Kent CK, et al. Household transmission of 2009 pandemic influenza A (H1N1) virus in the United States. N Engl J Med. 2009;361:2619–27. PubMed http://dx.doi.org/10.1056/NEJMoa0905498

9. Ferguson NM, Cummings DA, Cauchemez S, Fraser C, Riley S, et al. Strategies for containing an emerging influenza pandemic in Southeast Asia. Nature. 2005;437:209–14. PubMed http://dx.doi.org/10.1038/nature04017

10. Ghani A, Baguelin M, Griffin J, Flasche S, van Hoek AJ, Cauchemez S, et al. (2009) The early transmission dynamics of H1N1pdm influenza in the United Kingdom. PLoS Curr Influenza. 2009; RRN1130.

11. Lessler J, Reich NG, Cummings DA, Nair HP, Jordan HT, et al. Outbreak of 2009 pandemic influenza A (H1N1) at a New York City school. N Engl J Med. 2009;361:2628–36. PubMed http://dx.doi.org/10.1056/NEJMoa0906089

12. Gilks WR, Richardson S, Spiegelhalter DJ. Markov chain Monte Carlo in practice. London: Chapman and Hall; 1996.
